# Translational framework for implementation evaluation and research: a critical approach to patient-centred equity design

**DOI:** 10.1186/s43058-025-00789-8

**Published:** 2025-11-05

**Authors:** Carl R. May, Alyson Hillis, Katja Gravenhorst, Cory D. Bradley, Elvin Geng, Kasey Boehmer, Katie I. Gallacher, Carolyn A. Chew- Graham, Kate Lippiett, Christine M. May, Rachel Smyth, Ellen Nolte, Fiona Stevenson, Alison Richardson, Frances Mair, Anne MacFarlane, Victor M. Montori

**Affiliations:** 1 Department of Health Services Research and Policy, London School of Hygiene and Tropical Medicine, 15-17 Tavistock Place, London, WC1H 9SH UK; 2 Center for Dissemination & Implementation Science, Northwestern University Feinberg School of Medicine, 633 N. Saint Clair St., Chicago, IL 60611 USA; 3 Center for Dissemination and Implementation Research, Institute for Public Health, School of Medicine, Washington University, 660 S. Euclid, St. Louis, MO 63110 USA; 4 Division of Nephrology and Hypertension, and Knowledge and Evaluation Research Unit, Mayo Clinic, Plummer Building, Fourth Floor, 200 First St. SW, Rochester, MN 55905 USA; 5 General Practice & Primary Care, School of Health and Wellbeing, University of Glasgow, Clarice Pears Building, 90 Byres Road, Glasgow, G12 8TB UK; 6 School of Medicine, Keele University, David Weatherall Building, University Road, Staffordshire, ST5 5BG UK; 7 School of Health Sciences, University of Southampton, Building 67, West Highfield Campus, University Road, Southampton, SO17 1BJ UK; 8 Southampton, UK; 9 Department of Health Services Research, London School of Hygiene and Tropical Medicine, 15-17 Tavistock Place, London, WC1H 9SH UK; 10 Research Department of Primary Care and Population Health, UCL, Rowland Hill Street, London, NW3 2PF UK; 11 WHO Collaborating Centre for Participatory Health Research With Refugees and Migrants, School of Medicine, University of Limerick, Limerick, Ireland; 12 Knowledge and Evaluation Research Unit, Mayo Clinic, Plummer Building, Fourth Floor, 200 First St. SW, Rochester, MN 55905 USA

## Abstract

**Background:**

The field of implementation research has recently seen much interest in equity, with a strong emphasis on recognising and responding to disparities in care. Recent studies highlight the role of macro-level processes that translate meso-level institutional behaviours to micro-level healthcare practices, and that are generative of health and care inequities. They emphasise challenges patient-centredness and underscore the need for justice-oriented intervention design to address disparities and promote equitable care.

**Aim:**

To develop a patient-centred and justice-informed approach to the design of complex healthcare interventions and innovations in service delivery.

**Method:**

Patient-centred Equity Design was developed in five stages. Sociological, public health, and implementation science theories explaining the generation of modifiable inequities were identified, and relevant explanatory constructs were extracted from them and organised into a determinant framework. Framework elements were then translated into (a) process models characterizing causal mechanisms of systemic inequities; (b) generative principles to guide equity- and patient-centred interventions and services; and (c) critical design questions to appraise the ways that inequities are embedded in healthcare interventions and services.

**Results:**

Development work led to a determinant framework linking macro-level processes to meso- and micro-level healthcare inequities, and these were visualized in process models. The framework informed principles for the promotion of equitable, patient-centred interventions: fostering civility and dependability, ensuring clarity and continuity, and reducing workload and complexity. Four critical questions address relational inequalities, participation barriers, role expectations, and restitution for inequities. These were translated into proposed content for a simple appraisal tool to support the equitable design and evaluation of healthcare interventions and services.

**Conclusion:**

Patient-centred Equity Design integrates sociology, social justice, and implementation science to create equity-focused healthcare interventions. It offers a determinant framework, process models, generative principles, and critical questions to guide design. While not a validated tool, it enhances intervention development and service delivery, with potential for future Medical Research Council Framework integration. Patient- centred Equity Design provides actionable generative design principles to centre patient and caregiver experiences within intervention development, emphasizing restitution for inequities.

Contribution to the literature

- Patient-centred Equity Design is an approach to intervention design that explicitly addresses systemic mechanisms perpetuating healthcare disparities.
- Patient-centred Equity Design provides actionable generative design principles to centre patient and caregiver experiences within intervention development, emphasizing restitution for inequities.
- Patient-centred Equity Design offers critical evaluative questions that operationalise social justice theory, filling a significant gap in the implementation science literature.
- Patient-centred Equity Design advances implementation research by connecting structural determinants of inequity with practical intervention design strategies, promoting accountability in equity-focused healthcare delivery.

## Background

Questions about patient-centredness and social justice call on us to reflect on the problem of inequity. They are at the heart of critical accounts of healthcare [1]. Increasingly, they are at the heart not just of implementation science as a field of research and development [2–5], but also of improvement science [6, 7], and prevention science [8, 9]. These questions are found at the heart of debates about what patient-centredness and social justice can mean for large-scale services, in complex organisational settings [10], as well as the immediate relationship between practitioner and patient [11]. The fundamental problem that we must acknowledge in this paper is that disparities, inequalities, and inequities are not natural phenomena like gravity or tides. They are often designed into healthcare interventions and implementation processes, and are actively produced, implemented, and reproduced across services.

Healthcare services are not politically and socially neutral. They are produced and sustained within structured strategic action fields [12], in which macro-level actors shape the rules, resources, and relationships that govern practice at a micro level [13]. Power is central to this process of structuration: it shapes both the design of interventions and the contexts into which they are introduced [14]. Implementation processes give concrete form to these dynamics by translating the strategic intentions of one group of actors, those who propose interventions and innovations in service delivery—into the everyday practices of those who mobilise them [15]. In doing so, they reproduce not only the politics of healthcare systems, but also of the wider knowledge economies in which they are set. Because health service interventions and the implementation processes through which they are realised are inherently political, they encode assumptions about normative expectations of patients, caregivers, and practitioners. These assumptions are most visible in the treatment of minoritised and marginalised groups [16], whose participation in care is framed by processes of categorisation and stratification [17, 18]. These processes embed power relations into the design, delivery, and evaluation of services. They shape the work of patient and caregiver participation in healthcare, and the treatment burdens and administrative burdens that they must shoulder [19–21] as they do so. These processes of structuration are made visible in the design and organisation of services, and in the framing and implementation of health policies. They have powerful effects on the lived experience of patients and caregivers. The concept of *devitalisation*, introduced by Reynolds [17], is particularly relevant here. Reynolds highlights how macro-level structural and relational inequalities—manifest in bureaucratic demands, institutional indifference, or rigid care pathways—diminishes patients’ and caregivers’ energy, agency, and effectiveness, at the micro-level. These effects are especially acute for those already marginalised, as they must navigate services that exhaust rather than support them.

Sociological research on the work of participation in healthcare has consistently shown that it is institutionally constructed [15]. It is stratified, with different groups expected to contribute in different ways—and with varying levels of institutional support or recognition. Healthcare providers must critically examine how their expectations of patient and caregiver participation are constructed and operationalised. Doing so requires attention not only to what participation entails, but from whom participation is expected, under what conditions, and with what consequences for compliance/adherence and non-compliance/nonadherence. Only then is it possible to identify and promote actions that enable more equitable forms of agentic engagement.

What would a patient-centred and equity-informed way of thinking about complex interventions, and innovations in service design, in implementation science, and in other areas of health services research, look like? In this paper, we propose an approach to patient- centred and justice-oriented intervention: the Patient-centred Equity Design approach. This offers ways of thinking through the implications of inequities and promoting restitution for them: a set of tools to support patient-centred and equity-informed intervention and service design and development, and a theoretical approach that identifies the conditions and contexts in which they may be applied. Thinking about the design and implementation of complex healthcare interventions and innovations using this approach can help us bridge the gap between two important questions about the problem of patient-centredness in contemporary healthcare, proposed by Montori [22]. He asks, how can we best care for *these* patients (through population-based health improvement); and how can we best care for *this* patient (through patient-centred individualised care)?

Responding to Montori’s questions requires us to rethink the ways that complex interventions and service innovations are conceptualised and designed. Our approach in this paper is informed by Asad’s notion of prefigurative design [22]. This offers an approach to understanding that complex healthcare interventions are not just technical solutions to policy and practice problems. Instead, they realise political ideas about the nature of care. It draws attention to how institutional designs *prefigure* certain kinds of patients, clinicians, and relationships. In healthcare, this invites critical reflection on how interventions implicitly structure assumptions about what can be achieved through intervention and service design. This approach is consistent with social justice theory [23], an approach to intervention and service design that anticipates and actively works toward more just and equitable interventions.

The approach that we offer in this paper could contribute to this shift by offering a set of theory-informed tools grounded in patient-centredness and social justice. Amongst others, the Theoretical Domains Framework [23], the Consolidated Framework for Implementation Research [24], the PROLIFERATE framework [25], and Kahn and Moore’s equity questions [26], all raise questions of equity. In this context, we have sought to develop an approach to Patient-centred Equity Design. So, in this paper we seek to fill a gap in implementation science literature by integrating theories of inequality, implementation, user experience, and justice to support design practices that are explicitly oriented to the ways that health systems and structures are actively generative of inequities. We propose a reorientation of design practice—one that centres the situated knowledge of patients and caregivers and actively works to realise justice within intervention and service design. Because key terms are often used interchangeably in debates about disparities and inequalities, a glossary is provided (see Table 1) to clarify their use in this paper.

**Table 1:** Glossary

Complex intervention	An intervention characterised by many interacting components that target different behaviours and expertise, across multiple contexts [27]
Health Disparities	A particular type of difference in health in which disadvantaged social groups persistently experience social disadvantage or discrimination and thus worse health or greater health risks than more advantaged social groups [28]
Health Inequalities	Avoidable and unjust differences in health between individuals or populations [29]
Health Inequities	The presence of unfair and avoidable or remediable differences in health among social groups [29]
Implementation Framework	An analytic device to support, analyse or evaluate implementation processes. Normally composed of a taxonomy of theoretical constructs or empirical observations drawn from different sources [30]
Middle-range theory	A theory that is ‘sufficiently abstract to be applied to different spheres of social behaviour and structure’ but does not offer a set of general laws about behaviour and structure at a societal level. Its scope is defined by a limited set of assumptions from which can be derived hypotheses that may be confirmed or disconfirmed by empirical investigation [31]
Minimally disruptive care	A clinical method that seeks to operationalise patient-centredness whilst also seeking to reduce the personal workload involved in effective participation in care [32, 33]
Patient-centredness	An approach to care that emphasises the importance of patient (and caregiver) dignity, values, preferences, and capabilities, and which seeks to work from a holistic perspective [34]
Social Determinants	Politically modifiable causes of inequalities in health, that determine disparities between more and less advantaged groups [35]
Social Inequalities	Differences in health between different socioeconomic groups within a society [35]

## Methods

**Fig. 1:**
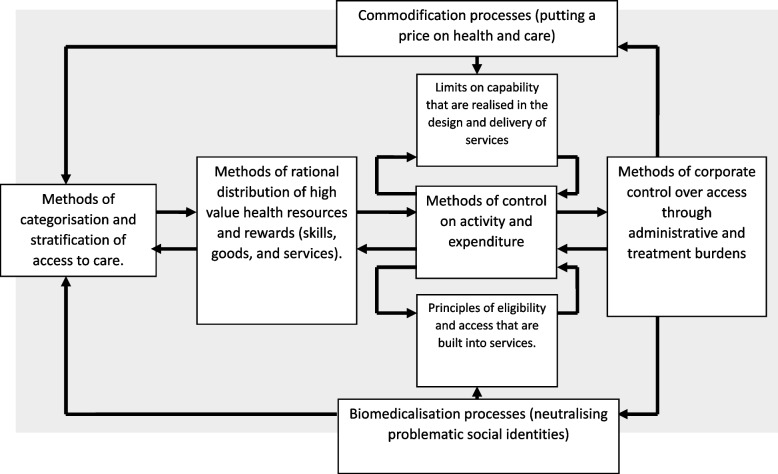
Process model: macro-level processes produce and reproduce structural inequities within healthcare systems

**Fig. 2:**
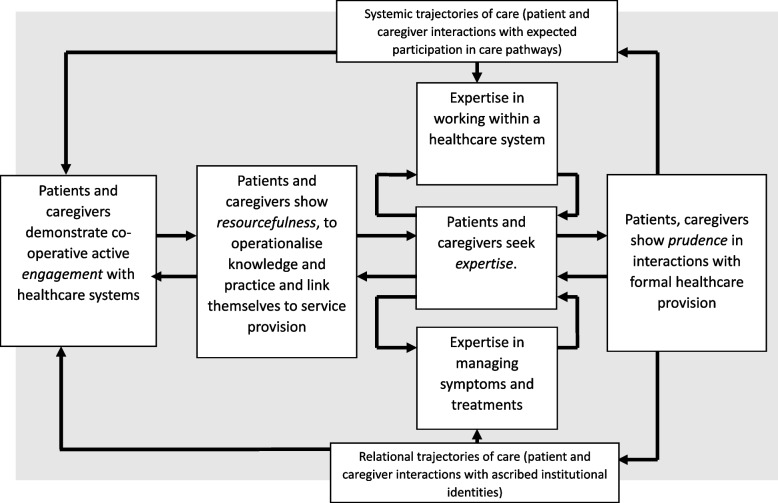
Process model: convergent forces shape normative expectations of patients

**Fig. 3:**
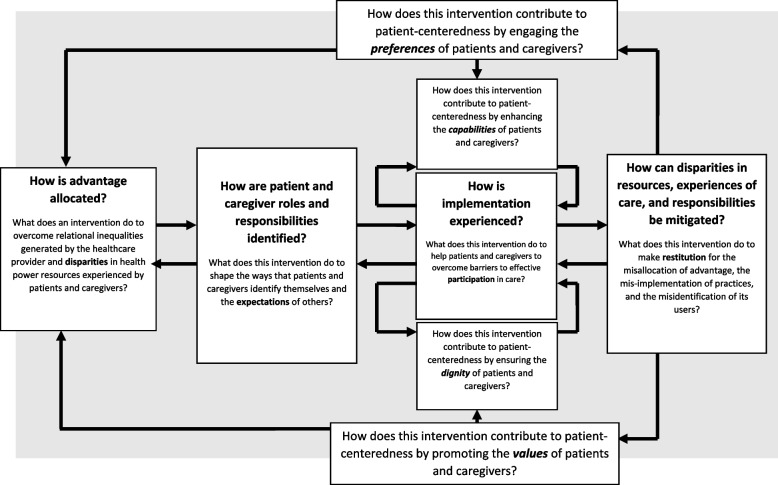
Patient-centred equity design: critical questions

The aim of the research reported in this paper was to develop a theoretically robust approach to (a) understanding the ways that inequities are generated and propagated within health services, and (b) translating that understanding into potential tools for intervention and service design. First, it is part of the development of a future Translational Framework for Implementation Evaluation and Research, (which develops and extends the application of Normalization Process Theory [36]). Second, it draws on the patient and public involvement contribution to a synthesis of 244 primary qualitative studies of lived experience of care in brain cancers, inflammatory bowel disease, bipolar disorder, schizophrenia, young onset dementia, and Parkinson’s disease, the EXPERTS II Study [37]. The general method used here reflected that successfully employed in the development of Normalization Process Theory [36]. It was undertaken in five phases.

i. Construct identification. Within the EXPERTS II Study [37] existing middle-range sociological and social justice theories were identified (see Table 2). Constructs of these theories had established explanatory utility for understanding inequalities in healthcare provision, the organisation of patient and caregiver work, and the shaping of illness trajectories [50].
ii. Constructing a determinant framework. Explanatory constructs from these theories were then extracted and arranged in a variable-by-variable matrix [51]. This matrix sets out theory-informed mechanisms that describes factors that shape patient and caregiver experiences within health systems (see Table 3). The matrix is consistent with Nilsen’s later definition of a determinant framework [30], in that it identifies factors implicated in the production and persistence of inequities in healthcare.
iii. Process Modelling. We then developed two process models to explain how these mechanisms operate across different levels of healthcare organisation and practice. These were constructed using an analytical approach to causal mechanisms [54], aimed at moving from description to explanation. The first model (see Fig. 1) illustrates how macro-level mechanisms give structure to inequitable patterns of care. The second (see Fig. 2) describes how expectations of patients and caregivers as workers are formed and reinforced within specific service contexts.
iv. Development of generative principles. Having developed a determinant framework and two process models, we translated these analyses into two design- oriented outputs. First, a set of generative principles [55] to inform the development of equity- and patient-centred interventions and service models. Second, a set of critical design questions oriented toward conceptual reframing, social justice, and restitution. These elements form the core of the PED design approach, summarised as a process model in Fig. 3.
v. Proposal of content for a simple appraisal tool. We consolidated the generative principles and critical questions into a qualitative investigation through implication analysis [59] providing content for a simple tool to appraise the potential production of inequities through intervention design and delivery. Examples of the use of the Patient-centred Equity Design for investigation are provided in Tables 4 and 5.

**Table 2:** Synopsis of middle-range theories underpinning the Patient-centred Equity Design approach

Theory	**Definition and Synopsis**
Strategic Action Field Theory	Explains how institutional fields are formed in which collective actors mobilise norms and values that stabilise the environments in which they operate, and provide a secure basis for goal-oriented outcomes [12, 38]
Relational Inequalities Theory	Explains how meso-level economic and social disparities are produced and reproduced within organisations. Identifies mechanisms through which organisations construct, produce, and legitimise material inequalities in the allocation of resources and rewards [39–42]
Health Power Resources Theory	Explains population-level disparities in power and resources. Identifies mechanisms that shape micro-level lived experience of health-related structural inequities, their stratification and distribution across populations, and their effects on the mobilisation of personal resources [18]
Burden of Treatment Theory	Explains mechanisms that motivate and shape the ways that the work of care and self-care can be delegated to patients and caregivers by healthcare providers. Situates agency within formal structures and informal networks that can frame capacity to effectively participate in care [43, 44]
Administrative Burden Theory	Explains how eligibility for, and access to, care is realised through different policy and bureaucratic systems. Shows how effective participation in care can be experienced as onerous, and has learning, compliance, and psychological costs for patients and caregivers [21]
Normalisation Process Theory	Explains mechanisms that motivate and shape implementation processes. Focuses on the social organisation of collaborative work and collective action through which strategic intentions can be realised and implementation processes can be accomplished [36, 45]
Sick role theory	Provides a framework for understanding societal, organisational, and professional constraints on patients and caregivers that frame the ways that they navigate and negotiate healthcare providers’ normative expectations of their beliefs and behaviours, and the ways that these are practically operationalised within social roles [46, 47]
Prefigurative Design	Provides a framework for translating values and aspirations for social justice into the design or material artifacts, human services, and organisational structures. Argues that interventions and service design are not neutral, natural, or inevitable but instead can embody injustices. Proposes a set of responses to these that can be incorporated into design [48]
Social Justice Theory	Provides a framework for understanding how historical processes and contemporary institutions create practices through which disadvantage is formed around race and ethnicity; sexuality, sex, and gender; disability, age, and other demographics. Proposes an overarching theory of justice through which inequalities and inequities can be identified, characterised and acted upon [49]

**Table 3:** Determinant framework—reproduction of modifiable inequities within healthcare systems

**Healthcare systems create inequitable contexts for care** [24, 30, 52, 53]	**Healthcare systems normalise inequities** [37, 41, 42, 54, 55]	**Healthcare systems embed inequities in social relations** [16, 56]	**Health professionals and administrators transfer inequities to patients and caregivers** [43, 44]	**Patients’ and caregivers’ perform the work of participation in care in inequitable circumstances** [46, 47]	**Health services can promote patient-centeredness and social justice *by design***	**Health services can become allies of patients and caregivers** [57, 58]
Controls on activity and expenditure leading to constraints on specific treatments or services; changes to the healthcare labour force; and redistribution of the burden of work.	Methods of stratifying access to care lead to the categorisation of problem populations.	Implementation of interventions and services bring about relational change in the ways services are organised.	Real and virtual relations are organised in ways that define participants’ assumed capabilities.	Patients and caregivers are expected to demonstrate active engagement in their interactions with health systems, providers, and practitioners.	Interventions and services that are respectful of providers and users represent moves towards civility.	Enhancing the capabilities of patients and caregivers.
The biomedicalisation and commodification of healthcare leading to large-scale, complex, task-allocated, and process-oriented services, and inflexible pathways through care.	Uneven distribution of high value resources and rewards lead to the inequitable allocation of skills, goods, and services.	Implementation of interventions and services brings about the creation of value as they undergo normalisation in practice.	Logics of practice are produced through formal and informal agreements that give authority to participants, and assign meaning to their actions.	Patients and caregivers are expected to show resourcefulness and to link themselves to changing patterns of service provision.	Interventions and services designed to eliminate opportunities for systemic failure represent moves towards dependability.	Identifying and acting upon the preferences of patients and caregivers.
Epidemiological, demographic, and socio-technical challenges leading to the strategic expansion of the health and social care sector.	Principles of eligibility lead to controls on access to the intervention or service.	Implementation of interventions and services bring about performative change in the ways that work is done, over time and across settings.	Ensembles of beliefs, behaviours, and material practices, are embedded in objects and procedures.	Patients and caregivers are expected to seek expertise, and to possess condition and service specific knowledge.	Interventions and services that encourage clarity of purpose, ease of navigation, and continuity of care represent moves towards simplicity.	Routinely incorporating the values of patients and caregivers into practice.
The obdurate corporate structures of health and social care services lead to the multiplication of administrative and treatment burdens for patients and caregivers.	Structural capabilities in health services lead to the realisation of disparities in the design and delivery of interventions and services.	Implementation of interventions and services bring about normative change, by modifying the rules and resources that govern action.	Formal and informal changes in norms and roles, information and material resources, shape participants’ delegated accountabilities.	Patients, caregivers, and practitioners are expected to demonstrate prudence to minimise the calls they make on formal healthcare provision.	Interventions and services that reduce workload, and restore agency and capacity to patients, caregivers and professionals represent moves towards the reduction of burden.	Ensuring the dignity of patients and caregivers.

**Table 4:** Example (1): Using PED to identify systemic sources of inequity

	**Bacchus et al. **[32]** support women at risk of intimate partner violence in the Palestinian Territories on the West Bank of the River Jordan**	**Parroche-Escudero et al. **[33]**: mainstreaming health equity within an organisation supporting research infrastructure in the UK**	**Bühler et al. **[60]**: improved access to care for carpal tunnel syndrome amongst Māori, Pasifika, low income, and rural populations in New Zealand**	**Siegler et al. **[45]**: improved provision of primary care services for transgender people in Onterio, Canada**
1. To what extent does the intervention foster equitable civility and dependability in interactions characterised by power and knowledge imbalances?	Partially. Providers exhibited caution due to cultural norms and concerns about safety and retaliation, which influenced interactions. Power imbalances and providers’ concerns sometimes limited deeper engagement with women’s issues, suggesting partial achievement rather than complete fostering of equitable civility and dependability.	Partially. This dialogue fostered civility by sensitising stakeholders to health inequalities and encouraging mutual understanding. However, it was less clear from the article whether it directly addressed existing power and knowledge imbalances beyond sensitisation, suggesting partial success rather than comprehensive fostering of equitable civility and dependability.	Partially. While the study emphasised the importance of culturally responsive information and communication to improve patient-clinician interactions, explicit actions to directly address power and knowledge imbalances are limited. However, recognising and reducing implicit racism and bias was explicitly highlighted, suggesting partial fostering of equitable civility and dependability​.	Yes. The intervention explicitly promoted creating a safe, inclusive environment, emphasising culturally competent interactions and reducing stigma. It actively addressed knowledge gaps through provider education to balance power dynamics, fostering dependable and respectful interactions between providers and transgender individuals​.
2. To what extent does the intervention promote equitable clarity of purpose, ease of navigation through services, and continuity of care?	Partially. While the intervention included a clear care pathway for responding to domestic violence, actual implementation encountered obstacles. Many women chose not to utilise external referrals, reflecting cultural and structural constraints.	Partially. The initiative sought to embed clarity around the purpose of reducing health inequalities through structured assessments and transparent monitoring. However, the direct effect on continuity of patient care was not a primary focus and thus was not explicitly reported or demonstrated.	Yes. The intervention was explicitly designed to improve access to care for carpal tunnel syndrome. It emphasised the need for culturally responsive communication and structured care pathways.	Yes. The intervention clearly defined primary care for transgender patients, including medical management, hormone therapy, and counselling. By integrating clear guidelines and provider training, it explicitly promoted ease of navigation through healthcare services and aimed to ensure continuity of care in primary care.
3. To what extent does the intervention reduce workload and simplify system complexity for all service users?	No. The intervention increased workload complexity for clinic case managers and other health care providers because of competition from other work. Providers explicitly mentioned increased workloads and the difficulty of balancing domestic violence care with other clinical duties, indicating that the intervention did not simplify the system but rather added complexity​.	No. Rather than reducing workload, the intervention introduced new procedures, training, and mandatory assessments, potentially increasing complexity for professionals and researchers. Participants described additional bureaucratic burdens, suggesting increased rather than reduced workload and complexity.	Partially. The intervention identified existing workload burdens, such as bureaucratic processes for accessing care and managing information scarcity. While the intervention aimed to simplify system navigation through improved care pathways participants described significant complexity in navigating insurance claims within resource-limited public care systems.	Partially. The intervention emphasised normalising transgender healthcare within existing primary care workflows, aiming for easy integration into routine practice. It acknowledged ongoing training needs and complexity around specialised aspects of transgender care, suggesting partial success in reducing overall system complexity​.
4. Is the intervention explicitly designed to address relational inequalities within healthcare organisations and disparities in health-related power between providers, patients, and caregivers?	Partially. The intervention intended to address domestic violence within healthcare through training providers and introducing referral pathways, implicitly addressing relational inequalities. However, it could not challenge the deeper structural or organisational power disparities in the healthcare system itself.	Yes. The intervention explicitly aimed to address relational inequalities by mainstreaming health equity across organisational culture, processes, and practices. It recognised and sought to transform structural biases, making health equity a shared responsibility for a range of organisational actors. This explicitly linked to reducing disparities in health-related power​.	Yes. The intervention explicitly targeted relational inequalities and aimed to address health equity by focusing on the experiences of Māori, Pasifika, low-income, and rural populations. By embedding culturally responsive communication and directly confronting systemic biases (such as racism and economic disparities), it explicitly addressed relational inequalities.	Yes. It explicitly targeted inequalities by training providers to recognise and address the particular healthcare needs experienced by transgender patients. Creating inclusive environments and educating practitioners explicitly addressed power disparities, aiming to normalise transgender care within primary care​.
5. Is the intervention designed to support patients and caregivers in overcoming barriers to meaningful participation in care?	Partially. Clinic case managers offered crucial emotional support and confidentiality, facilitating women’s participation in the intervention. However, external barriers—such as restrictions on movement, cultural stigma, fear of retaliation, and economic dependence—limited women’s meaningful participation in external support services.	Partially. The intervention emphasised public involvement in research and evaluation, this indirectly supported participation. Its primary focus was on sensitising researchers and providers rather than directly equipping patients and caregivers to overcome participation barriers in clinical contexts.	Yes. By explicitly exploring and addressing barriers such as poor-quality communication, inadequate information, and difficulties accessing services, the intervention supported meaningful patient participation. Efforts to simplify processes to reduce logistical barriers clearly aimed at supporting patient participation among marginalised populations.	Yes. By providing inclusive spaces, educating healthcare providers, and actively addressing discrimination, lack of knowledgeable providers, and inappropriate clinical environments, the intervention clearly supported transgender individuals in meaningful participation in their healthcare journey​.
6. Is the intervention designed to support patients and caregivers in effectively managing the expectations placed on them by others?	Partially. The intervention supported women in coping with their circumstances by providing emotional support and counselling at the clinic. Providers and women engaged in subterfuge to manage familial and societal expectations safely.	Partially. The intervention emphasised valuing public contributions, thus implicitly supporting participants in managing expectations regarding their role in shaping research and practice. Explicit mechanisms for managing broader societal or healthcare-related expectations on patients and caregivers were not fully described, indicating partial rather than full achievement.	Partially. The intervention highlighted how social expectations and institutional processes placed significant burdens on patients. While the intervention aimed at reducing these burdens, the support for managing wider expectations was indirect, suggesting partial effectiveness in managing external expectations placed on patients and caregivers.	Partially. While the intervention significantly reduced expectations by normalising transgender healthcare within primary care, mechanisms supporting transgender patients in navigating broader societal or familial expectations were not discussed in detail. The primary focus remained on healthcare interactions rather than external expectations management​.

**Table 5:** Example 2: Responding to the problem of restitution

	**Bacchus et al. **[32]**: support for women at risk of intimate partner violence in the Palestinian Territories on the West Bank of the River Jordan**	**Parroche-Escudero et al. **[33]**: mainstreaming health equity within an organisation supporting research infrastructure in North-West England**	**Bühler et al. **[60]**: improved access to care for carpal tunnel syndrome amongst Māori, Pasifika, low income, and rural populations in New Zealand**	**Siegler et al. **[45]**: improved provision of primary care services for transgender people in Onterio, Canada**
Acknowledgment	Recognised that inequities were perpetuated by cautious or avoidant interactions due to cultural norms and safety concerns, which limited effective responses to domestic violence.	Clearly articulated how previous organisational practices and policies have maintained inequities, including barriers experienced by marginalised groups in accessing care and participating in research.	Explicitly acknowledged and documented the inequities perpetuated by the current complexities of health insurance claims processes, inadequate public funding, and experiences of racism in accessing care.	Acknowledged past failures in healthcare provision, specifically recognising that lack of training and knowledge contributed directly to inequitable care experiences for transgender individuals.
Redistribution	Allocated specific resources and training toward empowering providers to safely address domestic violence without reinforcing inequities.	Reallocated organisational resources to systematically prioritise equity assessments across all services, explicitly targeting historically underserved populations.	Directed additional funding toward public surgical services and telehealth infrastructures specifically designed to address inequitable care access among Māori, Pasifika, rural, and low-income groups.	Allocated additional resources explicitly towards transgender-focused primary care programs, including dedicated funding, practitioner education, and supportive infrastructure (e.g., gender-neutral spaces).
Inclusion	Involved survivors directly in redesigning culturally safe response mechanisms, ensuring these explicitly confront stigma and power disparities.	Intentional efforts to involve disadvantaged and traditionally excluded groups, especially through active public involvement in the research process. Advisors’ involvement aimed to ensure interventions were responsive to the needs and perspectives of disadvantaged communities.	Redirected healthcare resources explicitly toward improving timely access for groups disadvantaged by welfare processes or geographical isolation.	Actively involved transgender communities in continuous co-design of services, empowering them in decision-making processes to correct historic neglect and marginalisation.
Restoration	Engaged openly with community stakeholders to rebuild trust eroded by historical neglect or cautious withdrawal.	Recognized accountability and focuses on a formal, explicit equity mainstreaming strategy. Limitations were acknowledged, including difficulties in holding individual researchers fully accountable for integrating equity systematically into their projects.	Involved affected populations in designing and refining the intervention, addressing systemic misimplementation explicitly.	Systematically enhanced practitioner education on transgender health, ensuring providers were fully equipped to offer equitable, affirming care, thus repairing trust eroded by past inequities.
Accountability	Implemented monitoring frameworks to track equitable provider-patient interactions, explicitly capturing the experiences of marginalised women.	Implemented mechanisms to ensure marginalised groups re clearly and accurately represented in service planning and delivery decisions.	Ensured health insurance and other funding processes no longer systematically misidentified or undervalued conditions affecting marginalised groups.	Implemented standardised affirming protocols across healthcare documentation and interactions (e.g., preferred names and pronouns, gender-neutral records), explicitly rectifying historical misidentification and related harms.

## Results

### How implementation processes can embed inequities within health services: a determinant framework

How should we understand the relationship between macro- and micro-level mechanisms that shape inequity? In Table 3 we present a determinant framework that describes how social determinants of health inequalities are translated from macro-level processes (economic constraints; biomedicalization; commodification; demographic change; and corporate structuring)—through meso-level system behaviour (institutional logics, policy constraints, and resource allocation, practices of stratification)—to micro-level behaviours, and their realisation through everyday healthcare practices. Table 3 outlines structural and processual factors that contribute to the production and reproduction of inequalities within health services. In Fig. 1, we show how these macro and meso-level mechanisms interact with each other to create a loop of continuous translation through which inequities are produced and reproduced. Against this background, there is a longstanding body of research that demonstrates how health inequalities are shaped by political and economic processes that structure access to care, exposure to risk, and differential treatment [17, 39, 40, 61]. These structures act as social determinants of health—and more precisely, as *politically modifiable* determinants that can be changed.

For patients, caregivers, and practitioners, structural determinants of health are performed through their everyday encounters with healthcare organisations. One key site of inequity is the assignment of administrative and treatment burdens to patients and caregivers. These burdens are increasingly formalised and routinised, giving institutional structure to patients’ and caregivers’ participation in care processes; when such burdens are imposed unevenly, or without appropriate support, they become sources of inequality in themselves [57, 58, 62]. Participation in healthcare is therefore not a neutral act. It is shaped by expectations that reflect dominant norms about patient responsibility, autonomy, and compliance that themselves are shaped by social practices of categorisation [63]. These expectations are often grounded in narrow or idealised models of the ‘good patient’, which exclude or penalise those who cannot or do not conform to them [57, 58, 62]. Relations between the mechanisms that shape these experiences are described in a second, related process model in Fig. 2.

### Generative principles for patient-centred and social justice-oriented design

So far, we have set out a structural approach to exploring the ways that inequities are formed and propagated within health services. In this section of the paper, we propose a set of patient-centred, justice-informed generative principles [55] for the design of interventions and services [48, 49]. These principles are grounded in theory and oriented towards practical action. Generative principles are not prescriptive; rather, they are analytically coherent propositions that direct attention to the implications of the phenomena under investigation and offer potential pathways for explanation and transformation [64]. We set out three generative principles. These are informed by the literature on participatory co-design and co- creation [56, 65–69].

These principles respond to the recognition that patient-centred care is deeply contested in the context of increasingly industrialised and commodified healthcare systems [22, 32, 60]. While empirically grounded theories of patient and caregiver work provide important explanatory leverage, they are often difficult to operationalise in design processes. Importantly, centring these experiences does not mean simply including representatives of these groups in advisory roles. Rather, it calls for the creation of interactional spaces in which genuinely mutual engagement, coordinated action, and co-learning can take place. These must be structured around participatory principles that enable shared decision-making, respect different forms of knowledge, and redistribute authority within design processes [67]. The aim is not only to make services more inclusive, but to reconfigure services in line with principles of equity and justice.

- Design Principle 1: *Co-create and co-design interventions and services that promote equitable civility and dependability in interactions shaped by power and knowledge asymmetries.*

This principle is fundamental to the social contract of care. It draws attention to how structurally induced disparities translate into lived disadvantages in care. Transforming relations between patients, caregivers, professionals, and provider organisations begins with recognising the significance of both civility and dependability. Civility matters because respectful interactions affirm the moral and social value of democratic participation in care. A lack of civility signals disrespect and exclusion, undermining trust and eroding engagement. Dependability matters because patients and caregivers rely on services to deliver what is promised. When interventions are overly complex, fragmented, or unreliable, the possibility of receiving care becomes contingent and precarious. Investing in civility and dependability means rethinking how intervention components are structured and how they connect. It means designing systems that reduce the risk of breakdown and that support respectful, predictable participation for all users.

- Design Principle 2: *Co-create and co-design interventions and services that promote clarity of purpose, ease of navigation, and continuity of care.*

This principle emphasises the importance of making health systems comprehensible and accessible to those who use them. It challenges designers to focus on the cumulative effects of fragmented care pathways, inconsistent service delivery, and unclear roles for patients and caregivers. These problems are not just technical. They are the product of normative expectations placed on patients and caregivers—expectations about what they should know, how they should behave, and how they should move through systems. These expectations often reflect professional logics rather than users lived realities. Simplicity matters because it facilitates meaningful engagement. Clear purposes, navigable systems, and continuous care foster integration, reduce exclusion, and make services more usable for everyone, especially those already facing structural disadvantage. Figure 2 develops this logic by showing how micro-level processes give shape to expectations of participation in everyday service encounters.

- Design Principle 3: *Co-create and co-design interventions and services that reduce workload and minimise system complexity for all service users.*

This principle addresses the distribution of cognitive, emotional, and logistical labour between organisations, patients, caregivers, and professionals. When services are designed without paying attention to these burdens, they transfer work onto individuals who may have limited capacity to absorb it [32]. Reducing unnecessary workload is not only a matter of efficiency— it is about restoring agency. Excessive demands on patients and caregivers can undermine participation, especially when they already face precarious social or health conditions [33]. Similarly, overburdened professionals may struggle to deliver care equitably, inattentive to the unique demands for support that differently situated patients require to make the intervention work. Designing for reduced complexity and redistributed workload makes interventions more workable. It also makes services more just. Figure 1 grounds this in macro- level processes that structure institutions globally and shape the conditions in which health inequities are produced. Figure 2 shows how these processes are enacted at the micro level through expectations of how patients and caregivers participate in care.

### Critical questions about proposed or existing interventions and services

The Patient-centred Equity Design approach offers a structured way to examine how interventions and services may deny dignity, reinforce exclusion, or amplify inequities. It is built on research that has shown how health services can reproduce inequity and disparities in the lived experience of healthcare innovations. Here, Patient-centred Equity Design draws attention to how interventions affect the communities they are designed to serve, and how design processes can be oriented toward justice. To engage with this complexity, we propose a set of four critical questions.

- Critical Question 1: *What does a complex intervention or service innovation do to address relational inequalities within healthcare provider organisations and disparities in patients’ and caregivers’ access to health power resources?*

This question challenges designers to consider how their interventions enhance the capabilities of patients and caregivers. It focuses attention on how advantage is allocated— or misallocated—within service structures, and on whether new interventions reproduce or reduce existing disparities.

- Critical Question 2: *What does the intervention or service do to reduce barriers to effective participation in care?*

Effective participation requires more than opportunities—it requires support, recognition, and fit between interventions and users’ lives. This question prompts reflection on how interventions engage with the preferences, constraints, and social positions of patients and caregivers. Ignoring this risks *mis-implementation*—the introduction of practices that entrench, rather than address, injustice.

- Critical Question 3: *What does the intervention or service do to shape the responsibilities and roles of patients and caregivers identify and manage the expectations of others?*

Interventions and services do not simply serve passive users; they constitute and classify them. Patients and caregivers are defined through professional knowledge, institutional practices, and policy approaches. This question asks whether design processes genuinely reflect the values and perspectives of users, or whether they impose identities and responsibilities that reinforce inequity through *misidentification*.

- Critical Question 4: *What does the intervention or service do to make restitution for prior misallocation, mis-implementation, or misidentification?*

This final question turns attention to accountability. It asks whether interventions can be actively framed in ways that repair the harms that result from structural inequities and flawed implementation. Can the intervention restore dignity, redress imbalance, and reduce burden? Or does it create new systemic inequalities that deepen existing disparities?

These questions are intended to be used both retrospectively—to examine existing interventions—and prospectively—to inform the development of new ones. They centre patient and caregiver experiences and support the design of interventions and services that are both patient-centred and justice-oriented. They are summarised in a process model that identifies the underlying constructs and their relationships with each other (see Fig. 3).

### Patient-centred equity design: content for an equity appraisal tool

In the final stage of developing the Patient-centred Equity Design approach, we explored how these theoretical resources could be translated into practical tools to guide both the design and evaluation of complex interventions and service innovations. This work aimed to bridge theory and practice. Rather than debating abstract theoretical positions, our objective was to translate them into a set of simple, provocative questions that would help designers and implementers reflect on the equity implications of their interventions.

Approaching the design of complex healthcare interventions from an equity perspective is critical to ensuring that they do more than acknowledge disparities—that they actively challenge and mitigate them. Here, the Patient-centred Equity Design approach offers a structured set of critical questions designed to assess whether interventions align with justice- oriented goals and reflect the lived realities of patients and caregivers. In this section of the paper, we operationalise our generative design principles and critical questions, presenting them as preliminary content for a simple appraisal tool, that aims to support understanding whether proposed or existing interventions address the conditions that produce and sustain inequity in healthcare. The framework consists of seven guiding questions, grouped into three domains:

A. *Questions derived from generative design principles*
  1. To what extent does the intervention foster equitable civility and dependability in interactions marked by power and knowledge imbalances?
  2. To what extent does the intervention promote equitable clarity of purpose, navigability of services, and continuity of care?
  3. To what extent does the intervention reduce workload and simplify system complexity—for all service users, particularly those experiencing disadvantage?
B. *Questions derived from critical equity concerns*
  4. Is the intervention explicitly designed to address relational inequalities within healthcare organisations, and disparities in health-related power between providers, patients, and caregivers?
  5. Is the intervention designed to support patients and caregivers in overcoming barriers to meaningful and sustained participation in care?
  6. Is the intervention designed to support patients and caregivers in managing the expectations placed on them by others?
C. *Questions derived from the need for accountability and restitution*
  7. How does the intervention or service make restitution for structural and systemic inequities?
    a Does it explicitly acknowledge the existence and effects of inequity?
    b Does it redistribute resources, include disadvantaged groups in meaningful ways, restore equal standing to those previously marginalised, and hold designers and implementers accountable for these aims?

These questions are not intended as an evaluation checklist, but rather as a set of tools for qualitative reflection and iterative design. They are intended to support transformation, not just compliance. By framing intervention assessment around issues of relational inequality, power asymmetries, and justice, Patient-centred Equity Design moves beyond conventional implementation criteria to focus on the lived implications of interventions for those most affected by them. Tables 4 and 5 demonstrate the application of this framework. They present a qualitative secondary analysis of four interventions included in a qualitative evidence synthesis informed by Normalization Process Theory [45]:

(i) Bacchus et al. [70]. Supporting women at risk of intimate partner violence in the Palestinian Territories.
(ii) Bühler et al. [71]: Improving access to carpal tunnel syndrome care among Māori, Pasifika, low-income, and rural populations in New Zealand.
(iii) Parroche-Escudero et al. [72]: Mainstreaming health equity within a UK research infrastructure organization.
(iv) Ziegler et al. [73]: Evaluating primary care provision for transgender people in Ontario, Canada.

These case studies illustrate how Patient-centred Equity Design can be used to examine the extent to which interventions resist or reproduce systemic inequities. The tables provide a layered analysis of how power, participation, and resource distribution are configured and experienced within each intervention. This approach shifts evaluation away from descriptive summaries and toward transformative inquiry, identifying concrete opportunities for responding to—and redressing—inequity.

## Discussion

Theories and frameworks in implementation science are most useful when they provide tools for thinking, not simply hypotheses to test or checklists to complete. Patient-centred Equity Design is intended as such a tool. It supports critical reflection on how to promote patient- centredness and address structural inequalities in healthcare intervention and service design. Experiences of inequity—reflected in the design, quality, and outcomes of care—are central to these concerns. Disparities, inequities, and inequalities can be located across multiple axes of stratification, including socioeconomic status, race and ethnicity, disability, health status, sex and gender, age, and religion [29, 35]. A core premise of Patient-centred Equity Design is that such disparities are not simply background conditions. They are actively produced and normalised through implementation processes. As Snell-Rood et al. [74] have argued, these considerations must be integrated at every stage of intervention development and implementation. While social categories are sometimes treated as crude analytic tools, they represent complex, intersecting lived realities [63, 75].

The Patient-centred Equity Design approach acknowledges that politically modifiable determinants of health and wellbeing are translated and propagated through combinations of organisational, material, and conceptual practices. These processes structure the implementation landscape and shape the lived experience of care. The approach is not intended to be used in isolation. In response to calls for an inclusive implementation science [3–5], it can be used in partnership with any of the main implementation science frameworks. Beyond this, it could add value to the implementation of the Medical Research Council framework for the development and evaluation of complex interventions [27]. That framework calls for more sophisticated understanding of complex interventions but does not confront inequity head-on. Its integration with Patient-centred Equity Design could drive patient-centred and equity-focused design into the mainstream.

### Patient and public involvement

Patient-centred Equity Design builds on findings from two programmes of work. First, a programme of work that seeks to integrate critical race theory and implementation theory as part of the development of the Translational Framework for Implementation Evaluation and Research (TRIPR). Second, a large-scale comparative qualitative evidence synthesis [50], which reviewed the lived experiences of patients and caregivers affected by brain cancers, inflammatory bowel disease, bipolar disorder, young-onset dementia, schizophrenia, and Parkinson’s disease. In the TRIPR programme and the EXPERTS II Study, members of informal patient advisory groups helped us by discussing their experiences of patient work and illness trajectories. Importantly, they talked about the ways that health professionals’ judgements about their illness were shaped by stigmatising views about illness identity, ethnicity, and religion. These judgements were sometimes experienced as frankly racist, as a result of which they asked to remain anonymous in this work [50]. Whilst patient and caregiver advisors reviewed the generative propositions on which the approach is based, they did not participate in the development of the content of the appraisal tool. Therefore, the next phase of development of Patient-centred Equity Design will involve intensive review and feedback from patients and caregivers as they become involved in intervention and instrument co- production.

### Strengths and limitations of patient-centred equity design

Patient-centred Equity Design bridges sociological research, implementation science, and design theory, producing a tool that appears to be both conceptually rich and practically applicable. It translates theories of inequality and justice into actionable generative principles and evaluative questions, through explicitly examining how interventions and services may reproduce or challenge inequities. Patient-centred Equity Design was developed through a systematic process, drawing on a large-scale qualitative evidence synthesis and incorporating established methods of theory extraction and causal modelling. Its process is replicable and auditable. Although grounded in healthcare research, Patient-centred Equity Design’s principles and critical questions are framed generically enough to be transferable to other human services contexts—such as social care, education, and justice—expanding its potential impact. Patient-centred Equity Design also has limitations. Although it is grounded in empirical data, it has not yet been applied prospectively to intervention co-design or implementation planning. Its practical utility in real-time service development remains to be tested. Patient-centred Equity Design also offers tools for qualitative implication analysis and interpretive judgement. In highly metric-driven policy environments, it may be perceived as less immediately actionable unless integrated with more conventional evaluation frameworks.

## Conclusion

Patient-centred Equity Design is a theoretically informed approach that responds to the challenge of designing patient-centred and equity-informed healthcare interventions. It is founded on middle-range theories in sociology, social justice, and implementation science. In this paper we have described the fundamental sources of Patient-centred Equity Design: a set of middle-range theories, a determinant framework, two process models, and a set of generative principles. We have also proposed a set of questions through which these can be applied in practice, and we have shown how their application can be performed. Although Patient-centred Equity Design can be applied to a wide variety of interventions, services, and contexts, it is not a validated research instrument. Instead, we propose it as a set of conceptual tools to inform the design of complex interventions and innovations in service delivery. We also propose its further development and integration in future revisions of the MRC Framework. Patient-centred Equity Design contributes to implementation science by offering a structured approach to intervention design with implications for how complex interventions and innovations in service design are made to work in the service of their users. Future research—and practical application—will clarify the strengths and limitations of its applicability.

## Data Availability

All data generated or analysed during this study are included in this published article.

## Abbreviations

EXPERTS II: Study acronym: *How are service user and caregiver participation in health and social care shaped by lived experiences of illness trajectories burdens of treatment and social inequalities? A theory-informed qualitative evidence synthesis*
MRC: Medical Research Council
PROLIFERATE: Study acronym: adaPtable fRamework tO evaLuate dIFfErent pRoducts of pArTicipatory rEsearch
TRIPR: Study acronym: *Translational Framework for Implementation Evaluation and Research*
